# Conflicting cerebrospinal fluid biomarkers and progression to dementia due to Alzheimer’s disease

**DOI:** 10.1186/s13195-016-0220-z

**Published:** 2016-12-09

**Authors:** Panagiotis Alexopoulos, Lukas Werle, Jennifer Roesler, Nathalie Thierjung, Lena Sophie Gleixner, Igor Yakushev, Nikolaos Laskaris, Stefan Wagenpfeil, Philippos Gourzis, Alexander Kurz, Robert Perneczky, for the Alzheimer’s Disease Neuroimaging Initiative (ADNI)

**Affiliations:** 1 Department of Psychiatry, University Hospital of Rion, University of Patras, 26504 Rio Patras, Greece; 2 Department of Psychiatry and Psychotherapy, Klinikum rechts der Isar, Technische Universität München, Ismaninger Straße 22, 81675 München, Germany; 3 Max Planck Institute of Psychiatry, Kraepelinstr. 2-10, 80804 München, Germany; 4 Department of Nuclear Medicine, Klinikum rechts der Isar, Technische Universität München, Ismaninger Straße 22, 81675 München, Germany; 5 Department of Informatics, Aristotle University of Thessaloniki, 54124 Thessaloniki, Greece; 6 Institute of Medical Biometrics, Epidemiology, and Medical Informatics (IMBEI), University of the Saarland, 66421 Homburg/Saar, Germany; 7 Neuroepidemiology and Ageing Research Unit, School of Public Health, Faculty of Medicine, The Imperial College of Science, Technology and Medicine, London, W6 8RP UK; 8 Department of Psychiatry and Psychotherapy, Ludwig-Maximilans-Universität München, Nußbaumstraße 7, München, 80336 Germany; 9 Department of Psychiatry, University Hospital of Patras, University of Patras, 26504 Rion Patras, Greece

**Keywords:** Prognosis, Alzheimer’s disease, Mild cognitive impairment, Cerebrospinal fluid

## Abstract

**Background:**

According to new diagnostic guidelines for Alzheimer’s disease (AD), biomarkers enable estimation of the individual likelihood of underlying AD pathophysiology and the associated risk of progression to AD dementia for patients with mild cognitive impairment (MCI). Nonetheless, how conflicting biomarker constellations affect the progression risk is still elusive. The present study explored the impact of different cerebrospinal fluid (CSF) biomarker constellations on the progression risk of MCI patients.

**Methods:**

A multicentre cohort of 469 patients with MCI and available CSF biomarker results and clinical follow-up data was considered. Biomarker values were categorized as positive for AD, negative or borderline. Progression risk differences between patients with different constellations of total Tau (t-Tau), phosphorylated Tau at threonine 181 (p-Tau) and amyloid-beta 1–42 (Aβ_42_) were studied. Group comparison analyses and Cox regression models were employed.

**Results:**

Patients with all biomarkers positive for AD (*N* = 145) had the highest hazard for progression to dementia due to AD, whilst patients with no positive biomarkers (*N* = 111) had the lowest. The risk of patients with only abnormal p-Tau and/or t-Tau (*N* = 49) or with positive Aβ_42_ in combination with positive t-Tau or p-Tau (*N* = 119) is significantly lower than that of patients with all biomarkers positive.

**Conclusions:**

The risk of progression to dementia due to AD differs between patients with different CSF biomarker constellations.

**Electronic supplementary material:**

The online version of this article (doi:10.1186/s13195-016-0220-z) contains supplementary material, which is available to authorized users.

## Background

An increasing body of evidence suggests that Alzheimer’s disease (AD) pathophysiology can be identified using biomarkers [1, 2]. AD is characterized by abnormal patterns in structural and functional imaging as well as by a pathological cerebrospinal fluid (CSF) signature [3]. The pathological CSF signature is defined by decreased CSF concentrations of the peptide amyloid-beta 1–42 (Aβ_42_) and increased levels of the proteins total Tau (t-Tau) and Tau phosphorylated at threonine 181 (p-Tau). It is of note that biomarkers reflect AD neuropathological changes with relatively high accuracy [4, 5]. In clinical practice, CSF biomarkers aid clinicians with decision-making, embody a key tool in the differential diagnosis especially of atypical dementia syndromes and increase diagnostic confidence [4, 6–9].

Biomarkers enable the identification of AD pathophysiology in pre-dementia stages of the disease, such as the stage of mild cognitive impairment (MCI) [10]. MCI is a clinical entity characterized by cognitive deficits which are so mild that activities of daily living remain largely unaffected [10]. It is a heterogeneous clinical syndrome with regard to aetiology, clinical appearance and prognosis. MCI can be caused by different diseases (e.g. AD, cerebrovascular disease, depression, frontotemporal lobar degenerations, etc.). As a consequence, only some patients with MCI progress to dementia due to AD or to other dementias within limited time periods, while cognitive functioning remains stable or even reverses to normal in others [11–15]. Biomarkers embody a valuable instrument in estimating the likelihood that MCI is engendered by AD. Interestingly, biomarkers are an integral part of the recently proposed National Institute on Aging–Alzheimer’s Association (NIA-AA) guidelines for the diagnosis of MCI in clinical research settings [16]. These guidelines suggest categorizing MCI according to the individual likelihood of underlying AD pathophysiology and the associated risk of developing AD dementia in the future; the highest likelihood category is characterized by biomarker findings pointing to the presence of AD pathophysiology, whereas the lowest likelihood category is characterized by findings not typical for AD. However, the criteria do not consider conflicting biomarker constellations, although they are very common in MCI [17–19].

A large number of studies have investigated the prognostic utility of established AD biomarkers (for instance [10, 20–28]), but none of them has taken into account the NIA-AA guidelines in combination with exclusive consideration of all three established CSF biomarkers. To shed light on this grey area, we conducted a retrospective study focused on differences in progression to dementia due to AD of patients with MCI and different CSF biomarker constellations.

## Methods

### Participants

The study sample encompassed data from all phases of the AD Neuroimaging Initiative (ADNI) (ADNI 1, Go and 2), a collaborative project of academic institutions and private corporations across the USA and Canada which began in October 2004. The study is coordinated by the Alzheimer’s Disease Cooperative Study at the University of California, San Diego. The ADNI data are disseminated by the Laboratory for Neuroimaging at the University of Southern California. Data used in this study were obtained from the ADNI database (www.adni-info.org) on 27 August 2014. Patients with MCI, fulfilling international diagnostic criteria [16], and with available CSF Aβ_42_, t-Tau and p-Tau values at baseline and clinical follow-up data were included. In the ADNI, patients with MCI had Mini-Mental-State Examination (MMSE) scores between 24 and 30, a Clinical Dementia Rating (CDR) score of 0.5, memory complaints and objective memory deficits on the Wechsler Memory-Scale-Logical Memory II test. They were not significantly impaired in their activities of daily living. Patients with diagnoses other than MCI at baseline, controls and patients with MCI but not all CSF biomarker findings available at baseline were excluded from the study. Patients diagnosed with AD dementia at follow-up met the NIA-AA diagnostic guidelines for dementia due to probable AD [1]. Regarding MCI patients who had not progressed to dementia but discontinued participation in follow-up visits or died, the data of their last follow-up visit were considered in the analysis.

### CSF collection and analysis

CSF collection, shipping, aliquoting, storage and analysis took place according to ADNI standard operating procedures (SOPs) [29]. It is noteworthy that some early CSF samples were mistakenly collected into inappropriate CSF collection tubes at the ADNI sites. However, this was corrected rapidly and the exposure time to any inappropriate CSF collection tube was of limited significance due to the short time the CSF was in contact with the transfer tubes (approximately 25.7 min) [29]. ADNI baseline CSF samples were analysed at the ADNI biomarker core laboratory at University of Pennsylvania according to published methods [5, 30]. CSF samples were put into the freezer at –80 °C. The CSF concentrations of Aβ_42_, t-Tau and p-Tau were measured using the multiplex xMAP Luminex platform with Innogenetics immunoassay kit-based reagents (INNO-BIA AlzBio 3; Ghent, Belgium) [30].

#### *APOE* genotyping

*APOE* genotypes were determined for all ADNI participants through analysis of blood samples using standard polymerase chain reaction methods [31].

### Classification of patients with MCI

In line with the NIA-AA algorithm, each patient’s biomarker values were categorized as either positive for AD, negative for AD or borderline. The definition of the range of borderline values was based on biomarker cut-off values and standard deviations (SDs) selected from previous reports on ADNI MCI patients [5]. The range of borderline values was specified with the aim to reach a reasonable compromise between minimizing the chance of an artificial categorization as positive and at the same time classifying less than 20% of the measured values of each biomarker as borderline. Values within 20% of the SD from the respective cut-off point were classified as borderline [32]. Aβ_42_ concentrations lower than the defined range of Aβ_42_ borderline values and t-Tau and p-Tau levels higher than the respective borderline ranges were assumed to be AD positive. All other biomarker values were considered negative. Aβ_42_, t-Tau and p-Tau concentrations <181 pg/ml, >105.2 pg/ml, and >26.6 pg/ml, respectively, were thus regarded as positive for AD. CSF levels of Aβ_42_ > 203 pg/ml and t-Tau and p-Tau concentrations <80.8 pg/ml and <19.4 pg/ml, respectively, were considered negative for AD.

Patients were classified according to their fluid biomarker profile into the following subgroups:

- MCI with no positive biomarkers (MCI_Non+_).
- MCI with all biomarkers positive (MCI_All+_).
- MCI with positive Aβ_42_ but negative or borderline p-Tau and t-Tau (MCI_Aβ+_).
- MCI with positive Aβ_42_ and positive p-Tau or t-Tau (MCI_Aβ+T+_).
- MCI with negative or borderline Aβ_42_ but positive p-Tau and/or t-Tau (MCI_T+_).

The biomarker constellations of the three latter subgroups are so far not being considered in the NIA-AA diagnostic guidelines, because for MCI patients with such biomarker constellations no likelihood grade for the presence of AD pathology is assigned by the NIA-AA criteria [16].

### Statistical analysis

The statistical analyses were performed in SPSS v19.0 for Windows (IBM Corp., Somers, NY, USA). Normal distribution of data was checked using the Kolmogorov–Smirnov test. The raw biomarker data of study participants were graphically presented by means of non-negative matrix factorization (NNMF) [32, 33], a data-learning technique particularly suited for analysing positive valued data so that the available information is condensed in a low-dimensional (2D) space. The overall set of measurements:

*X*_*i*_ = {Aβ_42_, t-Tau, p-Tau}unlikely_*i*_,  *i* = 1, 2, …, *N*_,_ where *N* is the total number of participants, was approximated as:

*X*_[*N*×3]_ ≈ *W*_[*N*×2]_ *B*_[2×3]_

in order to minimize the reconstruction error induced by the Frobenius norm: ||*X*-*WB*||^2^. In this way, the vector of measurements *X*_*i*_ associated with the *i*th participant took the form of:

*X*_*i*_ = *w*_*i*1_ *B*_1_ + *w*_*i*2_ *B*_2_,

where *B*_1_ and *B*_2_ were the unit length vectors for a parsimonious 2D representation and *w*_*i*1_ and *w*_*i*2_ were the corresponding components. Differences between the MCI subgroups regarding demographic and CSF data, MMSE scores, follow-up duration, presence of the *APOE* ε4 allele and progression rates to dementia due to AD were tested by analysis of variance (ANOVA), Bonferroni post-hoc analysis, Kruskal–Wallis test, Mann–Whitney test and chi-square test as appropriate. Differences in the hazard of progression between the MCI subgroups were analysed using Cox regression models, adjusting for patient characteristics that significantly differed between the subgroups. Two-sided *p* < 0.05 was considered statistically significant.

## Results

**Fig. 1:**
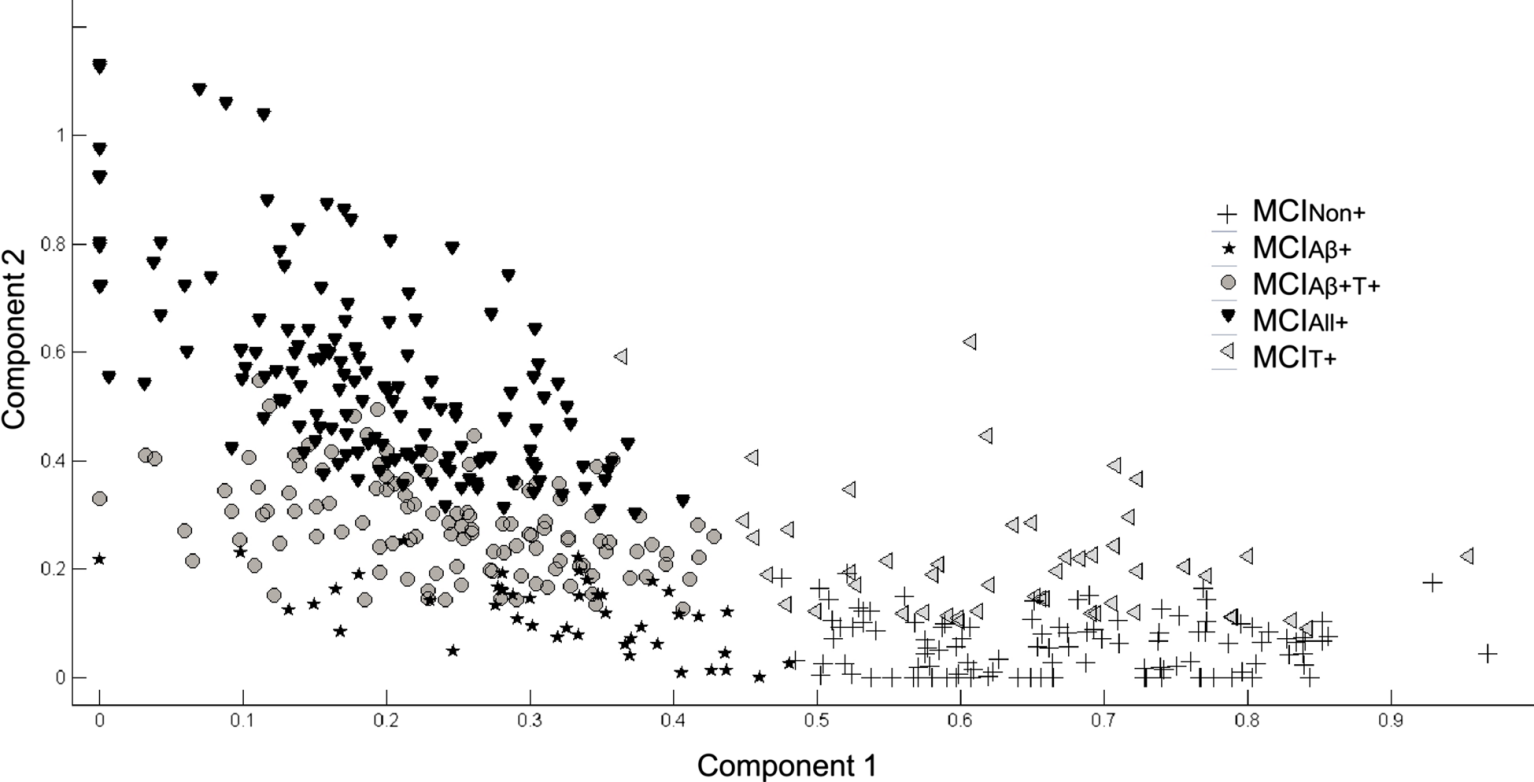
Condensed representation, as a 2D scatter plot, of the CSF biomarker values of the study participants. The ensemble of trivariate measurements of CSF Aβ_42_, p-Tau and t-Tau for all participants was analysed via NNMF and approximated by means of a bivariate data swarm that conveniently represents the total variation in the original data. Labels indicate the different groups and lend semantics to the plot. *MCI*_*Non+*_ MCI without positive CSF biomarkers, *MCI*_*Aβ+*_ MCI with positive Aβ_42_ and negative or borderline p-Tau and t-Tau, *MCI*_*Aβ+T+*_ MCI with positive Aβ_42_ and positive t-Tau or p-Tau, *MCI*_*All+*_ MCI with Aβ_42_ and both t-Tau and p-Tau positive, *MCI*_*T+*_ MCI with negative or borderline Aβ_42_ and at least p-Tau or t-Tau positive

A total of 469 MCI patients out of 1729 ADNI participants with available baseline data fulfilled the inclusionary criteria of the study. *APOE* ε4 and sex distribution, as well as age and MMSE scores, significantly differed between the subgroups (Table 1). In particular, the MMSE scores of the MCI_Non+_ subgroup were significantly higher compared with the scores of the MCI_All+_ (*p* < 0.001) and MCI_Aβ+T+_ (*p* < 0.01) subgroups. MMSE scores in the MCI_All+_ subgroup were significantly lower in comparison with those of the MCI_Aβ+_ (*p* = 0.02), MCI_Aβ+T+_ (*p* = 0.03) and MCI_T+_ (*p* < 0.01) subgroups. Across the five studied MCI subgroups, approximately 45% of patients had conflicting CSF biomarker constellations. Figure 1, a graphical presentation of participants’ Aβ_42,_ t-Tau and p-Tau CSF levels using NNMF, points to the high variability of the CSF biomarker findings in patients with MCI. Data were available from clinical follow-up visits conducted every 6 months up to 8 years after baseline. In total, 159 patients with MCI progressed to dementia due to AD. No patient progressed to any other form of dementia. The difference between the MCI subgroups in the proportions of patients who developed dementia due to AD within the follow-up period attained statistical significance (*p* < 0.001), whilst the duration of the follow-up period did not differ.

**Table 1:** Characteristics of the study sample

	MCI subgroup					*p* value
	MCI_Non+_	MCI_Aβ+_	MCI_Aβ+T+_	MCI_All+_	MCI_T+_	
*N*	111	45	119	145	49	
Age (years)	71.29 (7.83)	74.78 (6.59)	74.25 (7.06)	72.68 (7.40)	71.57 (9.00)	0.010
Education (years)	16.54 (2.70)	16.29 (3.07)	16.13 (2.77)	15.97 (2.83)	15.92 (2.86)	0.468
MMSE	28.13 (1.70)	27.64 (1.79)	27.42 (1.88)	26.93 (1.87)	27.92 (1.78)	<0.001
Sex (male:female)	67:44	34:11	78:41	73:72	29:20	0.020
*APOE* ε4 carriers (%)	21.62	42.22	62.18	77.93	26.53	<.001
CSF Aβ_42_ (pg/ml)	232.59 (30.25)	140.81 (26.14)	132.81 (23.70)	134.98 (20.96)	232.93 (30.24)	<0.001
CSF Aβ_42_ negative/borderline/positive for AD	87/24/0	0/0/45	0/0/119	0/0/145	39/10/0	<0.001
CSF p-Tau (pg/ml)	18.63 (4.44)	20.36 (4.45)	43.66 (16.00)	58.41 (2.53)	41.77 (13.58)	<0.001
CSF p-Tau negative/borderline/positive for AD	57/54/0	18/27/0	0/2/117	0/0/145	0/0/49	<0.001
CSF t-Tau (pg/ml)	50.66 (18.12)	55.53 (17.28)	78.37 (18.06)	158.09 (46.47)	74.86 (34.40)	<0.001
CSF t-Tau negative/borderline/positive for AD	106/5/0	42/3/0	59/58/2	0/0/145	29/15/5	<0.001
Follow-up period (months)	32.22 (23.34)	32.53 (23.50)	30.81 (22.34)	29.96 (21.01)	32.02 (11.64)	0.350
Dementia due to AD vs no dementia at follow-up	14:97	14:31	43:76	80:65	8:41	<0.001

Data presented as mean (standard deviation) or frequencies

*AD* Alzheimer’s disease, *MCI* mild cognitive impairment, *APOE* apolipoprotein E, *MMSE* Mini-Mental State Examination, *CSF* cerebrospinal fluid, *Aβ42* amyloid-beta 1–42, *p-Tau* tau phosphorylated at threonine 181, *t-Tau* total tau, *MCI*_*Non+*_ MCI without positive CSF biomarkers, *MCI*_*Aβ+*_ MCI with positive Aβ_42_ and negative or borderline p-Tau and t-Tau, *MCI*_*Aβ+T+*_ MCI with positive Aβ_42_ and positive t-Tau or p-Tau, *MCI*_*All+*_ MCI with Aβ_42_ and both t-Tau and p-Tau positive, *MCI*_*T+*_ MCI with negative or borderline Aβ_42_ and at least p-Tau or t-Tau positive

**Fig. 2:**
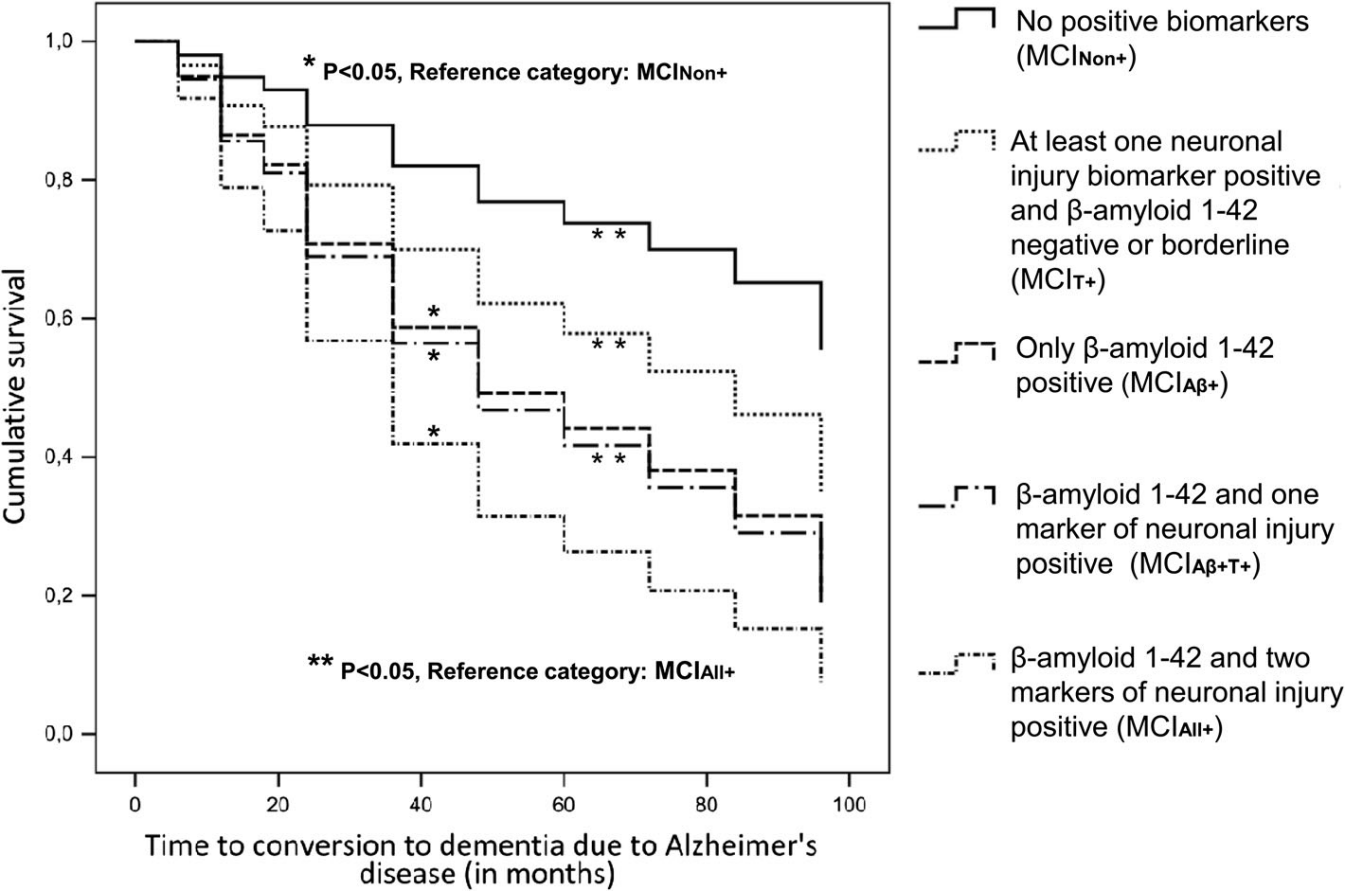
Cox regression survival curves for patients with MCI and different constellations of CSF Aβ_42_ and neuronal injury markers (p-Tau and t-Tau). *MCI*_*Non+*_ MCI without positive CSF biomarkers, *MCI*_*Aβ+*_ MCI with positive Aβ_42_ and negative or borderline p-Tau and t-Tau, *MCI*_*Aβ+T+*_ MCI with positive Aβ_42_ and positive t-Tau or p-Tau, *MCI*_*All+*_ MCI with Aβ_42_ and both t-Tau and p-Tau positive, *MCI*_*T+*_ MCI with negative or borderline Aβ_42_ and at least p-Tau or t-Tau positive

**Fig. 3:**
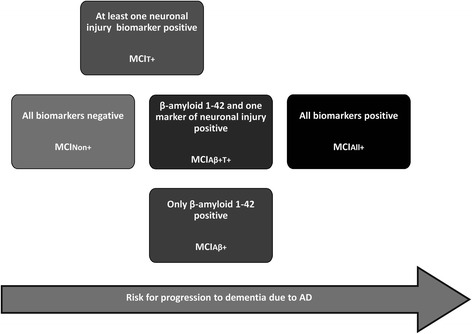
Risk for progression to dementia due to AD of patients with MCI and different constellations of CSF Aβ_42_ and neuronal injury markers (p-Tau and t-Tau). *AD* Alzheimer’s disease, *MCI*_*Non+*_ MCI without positive CSF biomarkers, *MCI*_*Aβ+*_ MCI with positive Aβ_42_ and negative or borderline p-Tau and t-Tau, *MCI*_*Aβ+T+*_ MCI with positive Aβ_42_ and positive t-Tau or p-Tau, *MCI*_*All+*_ MCI with Aβ_42_ and both t-Tau and p-Tau positive, *MCI*_*T+*_ MCI with negative or borderline Aβ_42_ and at least p-Tau or t-Tau positive

Cox regression analyses unveiled a significant association between group membership and risk of progression to AD dementia (*P* < 0.001), whilst sex, age and the presence of the *APOE* ε4 allele did not exert such an influence. As expected, lower MMSE scores were related to a higher hazard of developing dementia. The differences in progression risk between patients in the MCI_Non+_ subgroup and those with positive Aβ_42_ values (MCI_Aβ+_, MCI_Aβ+T+_, MCI_All+_ subgroups) reached statistical significance. In addition, the MCI_All+_ subgroup was at significantly higher risk for progression compared with both the MCI_Aβ+T+_ and MCI_T+_ subgroups. No further significant differences with regard to progression risk were detected between any other of the compared MCI subgroups (Table 2, Fig. 2). The risk pattern in relation to different biomarker constellations in MCI is presented in Fig. 3.

**Table 2:** Estimates of variables in Cox regression

Variable	Regression coefficient (*b*)	*p* value	Estimated hazard	95% confidence interval for hazard ratio
MCI subgroups		<0.001		
0 = MCI_Non+_*	0.992	0.009	2.697	1.279–5.686
1 = MCI_Aβ+_				
0 = MCI_Non+_*	1.060	0.001	2.887	1.538–5.422
1 = MCI_Aβ+T+_				
0 = MCI_Non+_*	1.481	<0.001	4.399	2.417–8.006
1 = MCI_All+_				
0 = MCI_Non+_*	0.591	0.185	1.806	0.753–4.330
1 = MCI_T+_				
0 = MCI_Aβ+_*	0.068	0.828	1.071	0.578–1.983
1 = MCI_Aβ+T+_				
0 = MCI_Aβ+_*	0.489	0.105	1.631	0.903–2.945
1 = MCI_All+_				
0 = MCI_Aβ+_*	–0.401	0.369	0.670	0.279–1.606
1 = MCI_T+_				
0 = MCI_Aβ+T+_*	0.421	0.029	1.524	1.045–2.222
1 = MCI_All+_				
0 = MCI_Aβ+T+_*	–0.469	0.234	0.625	0.289–1.355
1 = MCI_T+_				
0 = MCI_All+_*	–0.890	0.02	0.410	0.194–0.870
1 = MCI_T+_				
Age	0.007	0.536	1.007	0.985–1.030
Sex	0.021	0.905	1.021	0.727 – 1.434
0 = female*				
1 = male				
MMSE	–0.215	<0.001	0.807	0.738 – 0.882
*APOE* ε4	–0.331	0.072	0.718	0.501 – 1.030
0 = ε4 carriers*				
1 = ε4 non-carriers				

*Reference category

*MCI* mild cognitive impairment, *MCI*_*Non+*_ MCI without positive cerebrospinal fluid (CSF) biomarkers, *MCI*_*Aβ+*_ MCI with positive amyloid-beta 1-42 (Aβ42) and negative or borderline tau phosphorylated at threonine 181 (p-Tau) and total tau (t-Tau), *MCI*_*Aβ+T+*_ MCI with positive Aβ42 and positive t-Tau or p-Tau, *MCI*_*All+*_ MCI with Aβ42 and both t-Tau and p-Tau positive, *MCI*_*T+*_ MCI with negative or borderline Aβ42 and at least p-Tau or t-Tau positive, *MMSE* Mini-Mental State Examination; *APOE* apolipoprotein E

## Discussion

In line with a number of previous reports [17–19], but in contrast to others [8, 9, 34, 35], approximately half of the MCI cases in our study had conflicting CSF biomarker constellations. This discrepancy in the frequency of patients with conflicting CSF biomarker results could be possibly attributed to differences in study design. For instance, not all studies considered all three CSF AD biomarkers. In addition, past studies implemented a dichotomization strategy in interpreting biomarker findings, whilst in the present study biomarker values were categorized as positive, negative or borderline in line with the NIA-AA guidelines. Moreover, it is possible that academic, research centres recruit more patients with complex constellations of biomarker findings, whilst more patients with AD-typical CSF profiles and consequently more advanced neuropathology are recruited in non-academic, clinical settings. Interestingly, it has been shown that patients of a non-academic memory clinic suffered from more severe clinical symptoms in comparison with the patients of an academic memory clinic [36].

Constellations with conflicting CSF biomarker findings are not currently being considered by the NIA-AA criteria for MCI [16], and our study provides initial evidence on the role of conflicting CSF biomarker constellations for dementia risk estimation. Our findings indicate that compared with the constellation without positive biomarkers, the presence of Aβ_42_ positivity confers a higher risk for future AD dementia irrespective of t-Tau and p-Tau levels. Thus, it seems that Aβ_42_ is not only the first marker to become positive in the course of AD [37], but also the decisive marker to determine dementia risk. Despite the absence of general consensus, because a number of past reports point to a higher or almost similar prognostic utility of tau peptides compared with Aβ_42_ [20, 35, 38–42], our observations are in line with several previous studies which showed that Aβ_42_ has a higher prognostic utility in comparison with tau or failed to find an association between tau and cognitive deterioration [5, 22, 43, 44]. It is important to mention, however, that our results cannot necessarily be generalized to all patients with MCI, because the ADNI MCI cohort is deliberately limited to those with prominent memory deficits in order to enrich the sample with pre-dementia AD cases. MCI is heterogeneous by definition, and the studied biomarkers may react differently in early non-AD cases. Hence, the confounding effects of other brain pathologies associated with increased p-Tau and/or t-Tau levels (such as cerebrovascular changes, Lewy bodies, etc.) are minimized in ADNI, so that the role of Aβ_42_ as an indicator of AD pathophysiology may be exaggerated. Furthermore, it should be underscored that our findings could have been biased by the artificial definition of the range of borderline biomarker values as well as by the fact that, due to sample size reasons, borderline values were not considered separately from negative values in our analyses. Interestingly, an alternative analysis considering borderline and positive values together resulted in an amelioration of the significance of the role of Aβ_42_ (the results of the alternative analysis are presented in Additional file 1). Hence, further studies with larger samples enabling the separate consideration of borderline values are warranted before definite conclusions can be drawn.

The higher dementia risk in the MCI_All+_ subgroup compared with the other subgroups is in line with the proposed model of a temporal evolution of AD biomarkers as well as with a recently published, probabilistic, data-driven model of biomarker changes in sporadic AD, independent of a-priori patient staging and biomarker cut-off points [37, 45]. Our observation supports the assumption that as clinical symptoms advance and the threshold to dementia is reached, the abnormality of biomarkers becomes evident [46, 47]. The lack of significant difference in terms of dementia risk between the MCI_All+_ and MCI_Aβ+_ subgroups is probably a spurious finding, related to the relatively small size of the MCI_Aβ+_ subgroup and/or the definition of the cut-off values. This assumption is supported by the highly significant difference between the MCI_All+_ and MCI_Aβ+T+_ subgroups, although the latter subgroup has a higher dementia risk compared with the MCI_Aβ+_ subgroup as illustrated in Fig. 2. Moreover, the alternative analysis, in which borderline and positive biomarker values were treated as one group, unveiled a significant difference in progression risk between the MCI subgroup with all markers non-negative (positive or borderline) and the subgroup with only Aβ_42_ non-negative (Additional file 1). Thus, the observed lack of significant difference in dementia risk between the MCI_All+_ and MCI_Aβ+_ subgroups should be treated with caution. In addition, it is noteworthy that our results confirm the approach of the NIA-AA algorithm to assign the highest likelihood of AD to MCI patients with all biomarkers positive.

Our findings suggest that MCI patients with positive Aβ_42_ values are at the same risk for AD dementia whether or not they have one positive Tau marker (either t-Tau or p-Tau). Hence, patients with positive Aβ_42_ and non-positive or conflicting p-Tau/t-Tau levels, who cannot be categorized according to the current NIA-AA algorithm, seem to have the same dementia risk, which lies between that of the lowest and highest risk groups (MCI_Non+_ and MCI_All+_ respectively). However, this observation is in contrast with the findings of a large number of previous reports which have shown that MCI patients with two positive CSF markers have a higher risk to progress to dementia compared with MCI patients with only one positive biomarker [20, 22, 25, 28, 40, 44, 48–51]. As a result, this finding should be treated with caution. It cannot be precluded that the observation of the present study has been biased by the definition of the cut-off points and/or by the relatively small size of the MCI_Aβ+_ subgroup_._ Nonetheless, the alternative analysis in which borderline and positive biomarker values were treated as one group (non-negative values) did not reveal significant differences in the progression risk between the MCI subgroup with only Aβ_42_ non-negative and that with both Aβ_42_ and p-Tau or t-Tau non-negative (Additional file 1). Further studies are thus required in order to shed more light on the progression risk of the MCI_Aβ+_ and MCI_Aβ+T+_ subgroups.

In terms of dementia risk, MCI patients with one or two positive Tau markers but negative or borderline Aβ_42_ values (MCI_T+_ subgroup) may be placed between patients without positive biomarkers (MCI_Non+_ subgroup) and those with positive Aβ_42_ and non-positive or conflicting p-Tau/t-Tau levels (MCI_Aβ+_ and MCI_Aβ+T+_ subgroup, respectively). The term “suspected non-AD pathophysiology (SNAP)” has recently been proposed to designate individuals with abnormal markers of neuronal injury without evidence of amyloid accumulation [10, 18, 52]. According to our findings, the progression risk of the MCI_T+_ subgroup does not significantly differ from that of the MCI_Non+_ subgroup. Simultaneously, it is no different from that of the MCI_Aβ+_ and MCI_Aβ+T+_ subgroups. However, the dementia risk of the two latter subgroups does in fact differ from the MCI_Non+_ subgroup. As a consequence, the MCIT+ risk can be placed between that of MCI_Non+_ and MCI_Aβ+,_ MCI_Aβ+T+_ subgroups (Fig. 3). Nonetheless, this finding should also be treated with caution due to the limited size of the ADNI MCI_T+_ subgroup and because a prior study showed that the highest proportion of subjects who progressed to dementia was observed not only in the MCI subgroup with both amyloid and neuronal injury markers positive for AD but also in the MCI subgroup with only neuronal injury biomarkers positive [10]. This discrepancy could be explained by the different markers of neuronal injury considered in the two studies (neurochemical vs imaging), especially in light of the pathophysiological character of the former and the downstream topographical character of the latter [53].

The present study should be viewed in the light of some limitations. Owing to the lack of histopathological verification, the main outcome measure was based purely on clinical diagnoses, which are not always confirmed at autopsy [54]. Moreover, the ADNI encompasses individuals recruited at specialized research centres and does not mirror constellations in the community. This is clearly illustrated by the fact that within the follow-up period only conversion to dementia due to AD and not to other forms of dementia was observed. Furthermore, in building the MCI subgroups with distinct biomarker constellations we did not consider borderline values separately from negative values, because such an approach would have expanded the number of MCI subgroups and reduce their size. This limitation could explain the relatively high proportion—in comparison with previous reports—of patients in the MCI_Non+_ subgroup as well as in the MCI_T+_ subgroup who progressed to dementia due to AD [55], because it is possible that borderline biomarker values became positive shortly after baseline. In addition, it can be reckoned that our observations are biased by the artificial definition of the range of borderline biomarker values. In light of the lack of empirical data with regard to definitions of the range of borderline values, our findings should be treated with caution. Nonetheless, the NIA-AA guidelines clearly specify the presence of borderline biomarker values. As a consequence, further studies considering borderline biomarker values are warranted. A further shortcoming of the study is the collection of some early CSF samples into inappropriate tubes at the ADNI sites. Although the error was corrected rapidly and despite the relatively limited exposure time to any inappropriate CSF collection tube, this error could embody a source of bias because the use of different collection tubes increases intra-laboratory variability [34, 56–58]. Moreover, we did not take into account imaging biomarker data. However, it should be underscored that while combining imaging with neurochemical biomarker data may be relevant for research settings, it is rarely applicable to clinical settings because of limitations related to scanner equipment and sophisticated image analyses expertise.

## Conclusions

The present study provides a further piece of evidence for the prognostic differences between MCI subgroups with distinct neurochemical biomarker constellations. The study reveals significant differences between subgroups with conflicting biomarkers, on the one hand, and patients with all neurochemical biomarkers positive or non-positive (borderline or negative) for AD on the other. Even though our observations exclusively refer to neurochemical biomarkers and do not consider imaging markers, they point to the necessity of modifying/refining the NIA-AA algorithms for categorizing MCI.

## Additional files

Additional file 1: Presents the results of the alternative analysis based on consideration of borderline and positive biomarker values together. (DOCX 27 kb)
Additional file 2: Is an acknowledgements list for ADNI publications: ADNI infrastructure and site investigators. (PDF 661 kb)

## Abbreviations

AD: Alzheimer’s disease
ADNI: Alzheimer’s Disease Neuroimaging Initiative
ANOVA: Analysis of variance
APOE: Apolipoprotein E
Aβ_42_: Amyloid-beta 1–42
CDR: Clinical Dementia Rating
CSF: Cerebrospinal fluid
MCI: Mild cognitive impairment
MCI_All+_: Mild cognitive impairment with all biomarkers positive
MCI_Aβ+_: Mild cognitive impairment with positive Aβ_42_ but negative or borderline p-Tau and t-Tau
MCI_Aβ+T+_: Mild cognitive impairment with positive Aβ_42_ and positive p-Tau or t-Tau
MCI_Non+_: Mild cognitive impairment with no positive biomarkers
MCI_T+_: Mild cognitive impairment with negative or borderline Aβ_42_ but p-Tau and/or t-Tau positive
MMSE: Mini-Mental State Examination
NIA-AA: National Institute on Aging–Alzheimer Association
p-Tau: Phosphorylated tau at threonine 181
SD: Standard deviation
SNAP: Suspected non-AD pathophysiology
t-Tau: Total tau
