# Supplementary material for: Conflicting cerebrospinal fluid biomarkers and progression to dementia due to Alzheimer’s disease
Source: Alzheimers Res Ther. 2016 Dec 9;8:51. doi: 10.1186/s13195-016-0220-z (PMC5146856; doi:10.1186/s13195-016-0220-z)
Supplement: Additional file 1: — Presents the results of the alternative analysis based on consideration of borderline and positive biomarker values together. (DOCX 27 kb) [file 13195_2016_220_MOESM1_ESM.docx]

**Alternative analysis**

Due to sample size reasons borderline values were not considered separately. Here, the results of an analysis based on consideration of borderline and positive biomarker values together (non- negative) are presented. Patients were categorized according to their neurochemical biomarker profile into the following subgroups:

- MCI with all biomarkers negative(MCI_All-_)
- MCI with all biomarkers positive or borderline (non- negative) (MCI_Non-_)
- MCI with positive or borderline (non- negative) Aβ_42_ but negative p-Tau and t-Tau (MCI_2T-_)
- MCI with positive or borderline (non- negative) Aβ_42_ and negative p-Tau or t-Tau (MCI_T-_)
- MCI with negative Aβ_42_ and positive or borderline (non- negative) p-Tau and/or t-Tau (MCI_Aβ-_)

Table 1S. Characteristics of the study sample

|  | MCI subgroups | | | | | p- value |
| --- | --- | --- | --- | --- | --- | --- |
|  | MCI_All-_ | MCI_2T-_ | MCI_T-_ | MCI_Non-_ | MCI_Aβ-_ |  |
| N | 44 | 30 | 98 | 215 | 82 |  |
| Age  (years) | 70.84  (7.91) | 73.33  (7.62) | 73.95  (6.92) | 73.24  (7.35) | 71.33  (8.59) | P=0.028 |
| Education  (years) | 16.05  (2.80) | 16.30  (2.94) | 16.40  (2.77) | 16.08  (2.84) | 16.13  (2.84) | P=0.871 |
| MMSE | 27.68  (1.84) | 28.00  (1.64) | 27.63  (1.96) | 27.06  (1.84) | 28.28  (1.62) | P<0.001 |
| Sex (Male: Female) | 28:16 | 21:9 | 65:33 | 120:95 | 47:35 | P=0.29 |
| *APOEε4* carriers (%) | 20.93 | 33.33 | 53.06 | 72.56 | 19.51 | P<0.001 |
| CSF Aβ_42,_ (pg/ml) | 239.50  (23.35) | 164.12  (31.63) | 143.65  (32.32) | 136.25  (23.31) | 245.25  (25.57) | P<0.001 |
| CSF Aβ_42_ negative/ bordeline/ positive for AD | 44/0/0 | 0/12/18 | 0/15/83 | 0/7/208 | 82/0/0 | P<0.001 |
| CSF p-Tau, (pg/ml) | 15.13  (2.56) | 15.09  (2.64) | 35.01  (14.43) | 53.91  (21.32) | 31.16  (12.91) | P<0.001 |
| CSF p-Tau negative/ bordeline/ positive for AD | 44/0/0 | 30/0/0 | 0/34/64 | 0/7/208 | 1/42/39 | P<0.001 |
| CSF t-Tau, (pg/ml) | 44.50  (14.88) | 40.55  (13.43) | 61.88  (13.45) | 136.88  (49.24) | 65.52  (28.49) | P<0.001 |
| CSF t-Tau negative/ bordeline/ positive for AD | 44/0/0 | 30/0/0 | 98/0/0 | 0/67/148 | 64/14/4 | P<0.001 |
| Follow-up period (months) | 37.50  (26.84) | 31.80  (23.64) | 27.31  (18.62) | 30.56  (21.80) | 28.39  (19.35) | P=0.407 |
| Dementia due to AD vs. No dementia at follow-up | 7:37 | 5:25 | 26:72 | 110:105 | 11:71 | P<0.001 |

Data presented as mean (standard deviation) or frequencies.
MCI: Mild cognitive impairment; *APOE*: Apolipoprotein E; MMSE: Mini mental state examination; CSF: cerebrospinal fluid; Aβ42: amyloid β 1-42; p-Tau: tau phosphorylated at threonine 181; t-Tau: total tau; MCI_All-_: MCI with all CSF biomarkers negative; MCI_2T-_: MCI with positive or borderline Aβ_42_ and negative p-Tau and t-Tau; MCI_T-_: MCI with positive or borderline Aβ_42_ and negative t-Tau or p-Tau; MCI_Non-_: MCI with Aβ_42_ and both t-Tau and p-Tau positive or borderline; MCI_Aβ-_: MCI with negative Aβ_42_ and positive or borderline (non- negative) p-Tau and/or t-Tau

Table 2S. Estimates of variables in Cox regression

| Variable | Regression coefficient (b) | | p-value | Estimated hazard | 95% Confidence interval for hazard ratio |
| --- | --- | --- | --- | --- | --- |
| MCI subgroups |  | | <0.001 |  |  |
| 0= MCI_All-_*  1= MCI_2T-_ | | 0.269 | 0.648 | 1.309 | 0.412 – 4.155 |
| 0= MCI_All-_*  1= MCI_T-_ | | 0.743 | 0.088 | 2.103 | 0.895 – 4.944 |
| 0= MCI_All-_ *  1= MCI_Non-_ | | 1.307 | 0.001 | 3.695 | 1.681 – 8.122 |
| 0= MCI_All-_ *  1= MCI_Aβ-_ | | 0.086 | 0.859 | 1.090 | 0.420 – 2.828 |
| 0= MCI_2Τ-_*  1= MCI_T-_ | | 0.475 | 0.339 | 1.607 | 0.607 – 4.253 |
| 0= MCI_2Τ-_*  1= MCI_Νοn-_ | | 1.038 | 0.027 | 2.823 | 1.126 – 7.082 |
| 0= MCI_2T-_ *  1= MCI_Aβ-_ | | -0.183 | 0.735 | 0.833 | 0.289 – 2.401 |
| 0= MCI_T-_ *  1= MCI_Non-_ | | 0.563 | 0.010 | 1.757 | 1.143 – 2.699 |
| 0= MCI_T-_ *  1= MCI_Aβ-_ | | -0.657 | 0.077 | 0.518 | 0.250 – 1.073 |
| 0= MCI_Non-_*  1= MCI _Aβ-_ | | -1.221 | <0.001 | 0.295 | 0.154 – 0.567 |
| Age | | 0.006 | 0.555 | 1.007 | 0.985 – 1.028 |
| MMSE | | -0.212 | <0.001 | 0.809 | 0.740 – 0.883 |
| *APOE* ε4  0= ε4 carriers*  1= ε4 non carriers | | -0.309 | 0.092 | 0.735 | 0.513 – 1.052 |

MCI: Mild cognitive impairment; *APOE*: Apolipoprotein E; MMSE: Mini mental state examination; MCI_All-_: MCI with all cerebrospinal fluid (CSF) biomarkers negative; MCI_2T-_: MCI with positive or borderline amyloid β 1-42 (Aβ_42_) and negative tau phosphorylated at threonine 181 (p-Tau) and total tau (t-Tau); MCI_T-_: MCI with positive or borderline Aβ_42_ and negative t-Tau or p-Tau; MCI_Non-_: MCI with Aβ_42_ and both t-Tau and p-Tau positive or borderline; MCI_Aβ-_: MCI with negative Aβ_42_ and positive or borderline (non- negative) p-Tau and/or t-Tau; MMSE: Mini mental state examination; *APOE*:Apolipoprotein E; *: reference category.
